# First voltammetric analysis of two possible anticancer drug candidates using an unmodified glassy carbon electrode

**DOI:** 10.1038/s41598-024-68309-7

**Published:** 2024-07-27

**Authors:** Katarzyna Tyszczuk-Rotko, Katarzyna Staniec, Krzysztof Sztanke, Małgorzata Sztanke

**Affiliations:** 1 Faculty of Chemistry, Institute of Chemical Sciences, Maria Curie-Skłodowska University in Lublin, 20-031 Lublin, Poland; 2 Laboratory of Bioorganic Compounds Synthesis and Analysis, Medical University of Lublin, 4A Chodźki Street, 20-093 Lublin, Poland; 3 Department of Medical Chemistry, Medical University of Lublin, 4A Chodźki Street, 20-093 Lublin, Poland

**Keywords:** Two anticancer drug candidates, Differential pulse voltammetry, Glassy carbon electrode, Spiked urine samples, Electrochemistry, Physical chemistry

## Abstract

Dimethyl 2-[2-(1-phenyl-4,5-dihydro-1*H*-imidazol-2-yl)hydrazinylidene]butanedioate (DIHB) and 8-(3-chlorophenyl)-2,6,7,8-tetrahydroimidazo[2,1-*c*][1,2,4]triazine-3,4-dione (HDIT) are promising candidates for anticancer agents, the first analytical procedures of which are presented in this paper. The commercially available unmodified glassy carbon electrode (GCE) was used as a sensor for the individual and simultaneous differential pulse voltammetric (DPV) determination of these possible anticancer drugs. The findings concerning the electrochemical behaviour indicated that DIHB and HDIT display at GCE, as a sensor, the oxidation peaks at 1.18 and 0.98 V, respectively (vs. Ag/AgCl, 3.0 mol L^−1^ KCl) in the 0.125 mol L^−1^ acetate buffer of pH = 4.5, which were employed for their quantification. Various experimental parameters were carefully investigated, to achieve high sensitivity in voltammetric measurements. Finally, under the optimised conditions (*t* of 60 s, Δ*E*_A_ of 75 mV, *ν* of 225 mV s^−1^, and *t*_m_ of 2 ms), the proposed DPV procedure with the GCE demonstrated broad linear sensing ranges (1–200 nmol L^−1^—DIHB and 5–200 nmol L^−1^—HDIT), boasting the detection limits of 0.18 nmol L^−1^ for DIHB and 1.1 nmol L^−1^ for HDIT. Moreover, the developed procedure was distinguished by good selectivity, repeatability of DIHB and HDIT signals and sensor reproducibility. The practical application of this method was demonstrated by analysing the urine reference material without any prior treatment. The results showed that this environmentally friendly approach, with a modification-free sensor, is suitable for the sensitive, selective and rapid quantification of DIHB and HDIT.

## Introduction

**Figure 1:**
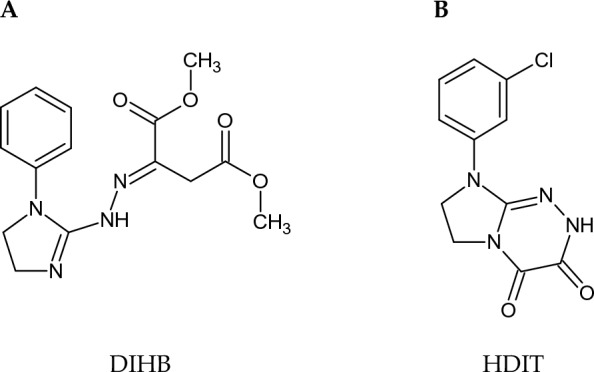
Structures of electroactive molecules that were studied: (**A**) dimethyl 2-[2-(1-phenyl-4,5-dihydro-1*H*-imidazol-2-yl)hydrazinylidene]butanedioate (DIHB), (**B**) 8-(3-chlorophenyl)-2,6,7,8-tetrahydroimidazo[2,1-*c*][1,2,4]triazine-3,4-dione (HDIT).

Among pharmacologically important heterocycles, a tautomeric imidazoline/dimethyl butanedioate hybrid and tetrahydroimidazotriazine-3,4-dione (Fig. 1A and 1B, respectively) with fully established molecular structures^1–7^, may be regarded as promising drug candidates because of their well-documented biological activity profile and advantageous properties^2–4,6,7^. Dimethyl 2-[2-(1-phenyl-4,5-dihydro-1*H*-imidazol-2-yl)hydrazinylidene]butanedioate (DIHB) (Fig. 1A) reveals antiproliferative effects in human tumour cells of the ovary, and effectively protects red blood cells from oxidative stress-induced haemolysis. This potential drug is less toxic to normal Vero cells compared to cancer cells, and non-toxic to erythrocytes^2^. In addition, DIHB possesses a detailed thermal characterisation. It is the most thermodynamically stable crystal form, which is unable to undergo any polymorphic transformations, is characterised by high chemical purity and high thermal stability^2^. In turn, 8-(3-chlorophenyl)-2,6,7,8-tetrahydroimidazo[2,1-*c*][1,2,4]triazine-3,4-dione (HDIT) (Fig. 1B) exhibits antiproliferative activity in human ovarian adenocarcinoma cells and human myeloma cells, is less toxic to two non-tumour cell lines (i.e., human skin fibroblasts and African green monkey kidney cells)^6^, is non-toxic to red blood cells (data not published), and is low in toxicity to mice^3,4^. Additionally, this compound reveals potent antinociceptive activity in reversing established acetic acid-induced hyperalgesia in mice, is thermally stable, does not undergo any polymorphic transformations and exhibits high chemical purity^3,4,7^. Due to favourable selectivity, both molecules (DIHB and HDIT) are promising candidates for further in vivo studies in the drug development process.

It is known from the literature that combination anticancer therapy is more effective than monotherapy. In addition, modern chemotherapy usually achieves better therapeutic results by combining more selective anticancer drugs from various classes, having different mechanisms of action^8^. Taking into account the potential usefulness of our drug candidates in combined cancer chemotherapy, it seems worthwhile to develop the first analytical method for detection and quantitative determination of electroactive DIHB and HDIT.

However, there is a research gap since no analytical procedure allowing the quantitative analysis of these small molecules has been elaborated to date. We are aware of this that the newly developed method—after evaluating its linearity, selectivity, sensitivity, detection and quantification limits—will have a chance to be used in clinical analytics. On the other hand, up to date, no information about the electrochemical behaviour of these molecules has been disclosed, although DIHB and HDIT contain electroactive moieties allowing their electrochemical detection and determination.

Chromatographic and spectroscopic methods are the most frequently used in the analysis of pharmacologically active compounds. However, they are labour-intensive, time- and reagents-consuming, and require an expensive equipment^9,10^. An alternative are electrochemical methods, including voltammetry, which are characterised by the sensitivity, the simplicity of the used procedures, and a short analysis time^11–27^.

Voltammetric sensors are widely used in the analysis of biologically active compounds on different matrices since they are characterised by many desirable properties such as an ease of manipulation, high sensitivity and satisfactory selectivity^11–15^. However, some of them require a complicated process of preparing the electrode itself and/or the use of various environmentally unfriendly solvents and reagents. Therefore, in our studies an unmodified glassy carbon electrode (GCE) as an environmentally friendly sensor of choice was applied. GCE, which enables the study of the redox behaviour of pharmaceutically and biochemically relevant molecules, is favourable in voltammetric applications because of its properties such as the chemical stability, electrical conductivity and inertness over a broad potential window and low cost^16–23,28–31^.

Our goal is to utilise a modification-free glassy carbon electrode to develop the first, simple, selective and sensitive voltammetric method for individual and simultaneous DIHB and HDIT determination, according to the principles of green chemistry. The presented results are of particular importance because such a simple method, using a reusable and environmentally friendly sensor, may be useful in the future for monitoring the concentrations of these potential anticancer agents in the urine samples of treated patients.

## Results and discussion

### The nature of DIHB and HDIT processes at the GCE

**Figure 2:**
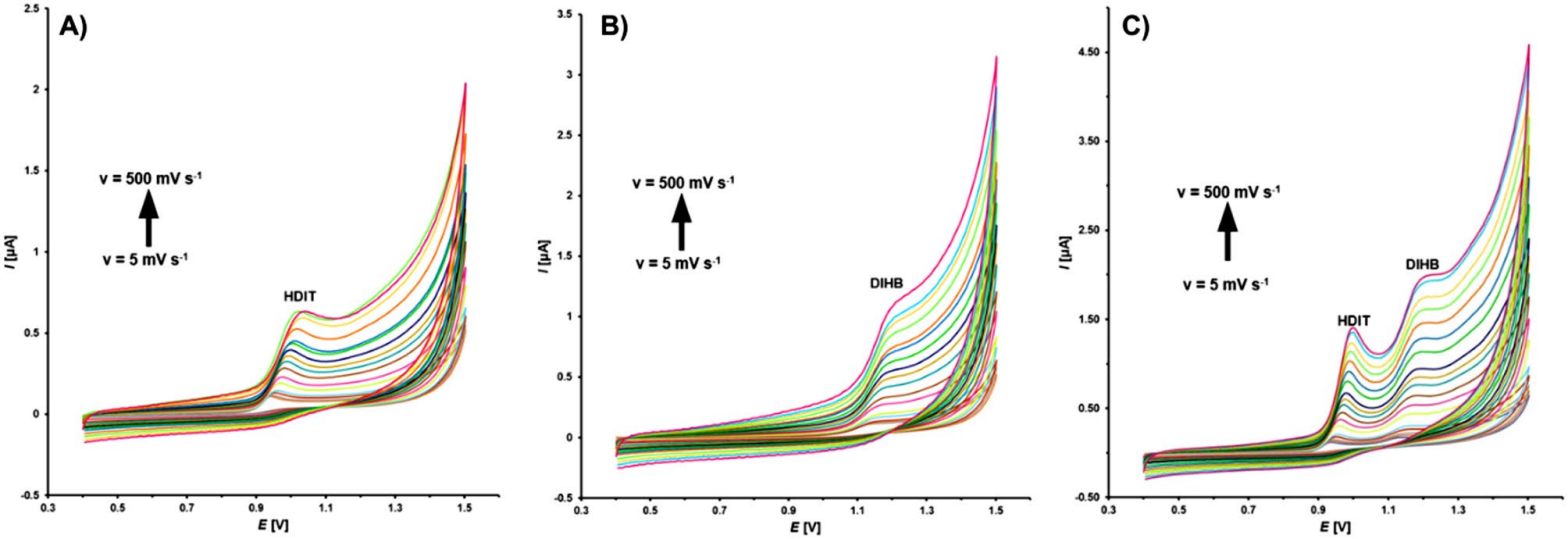
CV curves recorded at the GCE in a solution of 0.1 mol L^−1^ acetate buffer of pH = 4.5 containing 50 µmol L^−1^ HDIT (**A**), 50 µmol L^−1^ DIHB (**B**) or 50 µmol L^−1^ DIHB and HDIT (**C**) (the scan rate in the range of 5–500 mV s^−1^).

**Figure 3:**
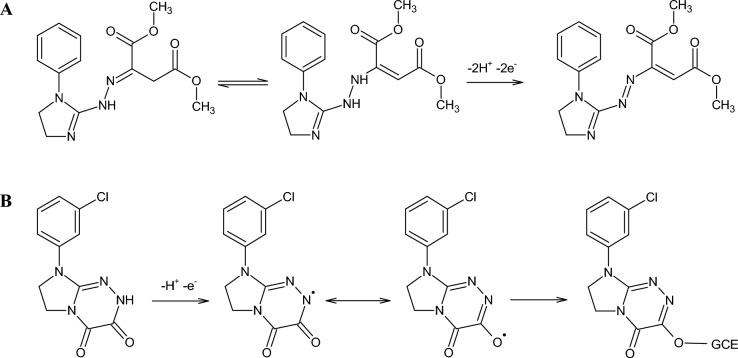
Hypothetical anodic oxidation pathways of DIHB (**A**) and HDIT (**B**).

The nature of DIHB and HDIT processes at the GCE for the individual and simultaneous determination was defined using the cyclic voltammetry (CV) at changing scanning rates (5–500 mV s^−1^) (Fig. 2). The oxidation of 50 µmol L^−1^ DIHB and 50 µmol L^−1^ HDIT in a solution of 0.1 mol L^−1^ acetate buffer of pH = 4.5 occurred in one step (${E}_{\text{p}}^{\text{a}}$: 1.18 V for DIHB, ${E}_{\text{p}}^{\text{a}}$: 0.98 V for HDIT, vs. Ag/AgCl, 3.0 mol L^−1^ KCl). One reduction peak of HDIT was observed at 0.90 V. The most likely mechanisms for the electrochemical behaviour of DIHB and HDIT are shown in Fig. 3. They were proposed taking into account our results from CV and DPV as well as previous experimental findings on some molecules with related moieties^32–36^. DIHB tautomerises to the hydrazo hybrid, which in a result of a two-electron oxidation process involving the loss of two protons forms the azo derivative (Fig. 3A). Support for this proposal came from previous experimental evidence showing that the oxidative dehydrogenation of hydrazoheteroaryl and hydrazoaryl structures is an effective electrochemical procedure for the synthesis of heterocyclic and aromatic azo compounds^32–34^. In turn, the amido form of HDIT undergoes one electron oxidation via abstraction of a proton from N2H to form (N2)^**⋅**^, existing in equilibrium in its (C3-O)^**⋅**^ resonance structure, which may be strongly adsorbed on the GCE surface (Fig. 3B). The hypothetical anodic oxidation pathway suggested for HDIT is similar to that disclosed for some imidazolidine-2,4-diones and nitrogenous bases of pharmaceutical relevance^35,36^.

**Figure 4:**
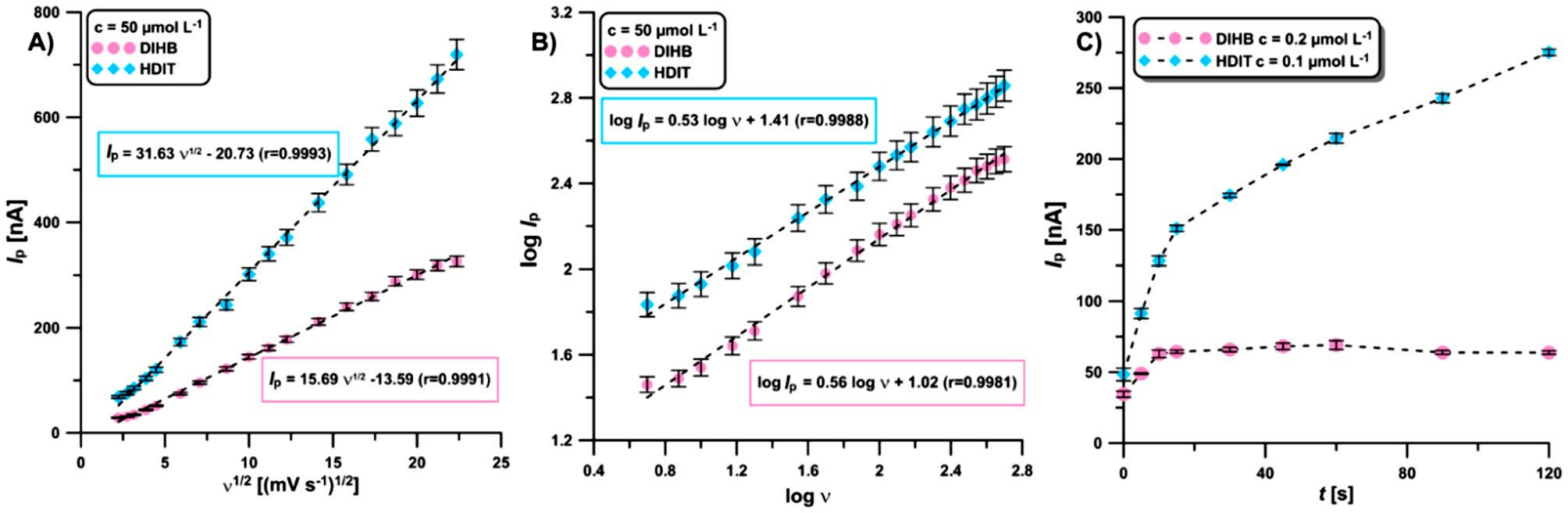
The relationship between *I*_p_ and *ʋ*^1/2^ as well as between log *I*_p_ and log *ʋ* for 50 µmol L^−1^ DIHB and HDIT (**A** and **B**). The solution mixing time (without applying the potential) on the DPV peaks of 0.2 µmol L^−1^ DIHB and 0.1 µmol L^−1^ HDIT (**C**). The average *I*_p_ values are given with standard deviation (SD, n = 3).

The CV experiments (0.1 mol L^−1^ acetate buffer of pH = 4.5 containing the 50 µmol L^−1^ DIHB and HDIT) at different potential scan rates (*υ*) allowed determining the relationship between *I*_p_ and *ʋ*^1/2^ as well as between log *I*_p_ and log *ʋ* for both analytes. The linear relationships between *I*_p_ vs. *ʋ*^1/2^ (Fig. 4A) and between log *I*_p_ vs. log *ʋ* (Fig. 4B) indicated a diffusion-controlled process for DIHB and HDIT^37^. For DIHB and HDIT oxidation, all curves showed r values close to unity, indicating that the electrooxidation of DIHB and HDIT occurs in a diffusional process. Therefore, the solution mixing time (without applying the potential) was examined on the DPV peaks of 0.2 µmol L^−1^ DIHB and 0.1 µmol L^−1^ HDIT. The mixing time was varied up to 120 s. As seen in Fig. 4C, the DIHB peak increases with increasing time up to 15 s and then becomes constant. In the case of HDIT, it increases over the entire tested range. As a compromise between the current intensity of both peaks, the mixing time of the solution before the measurement was 60 s.

### The DPV procedure optimisation

**Figure 5:**
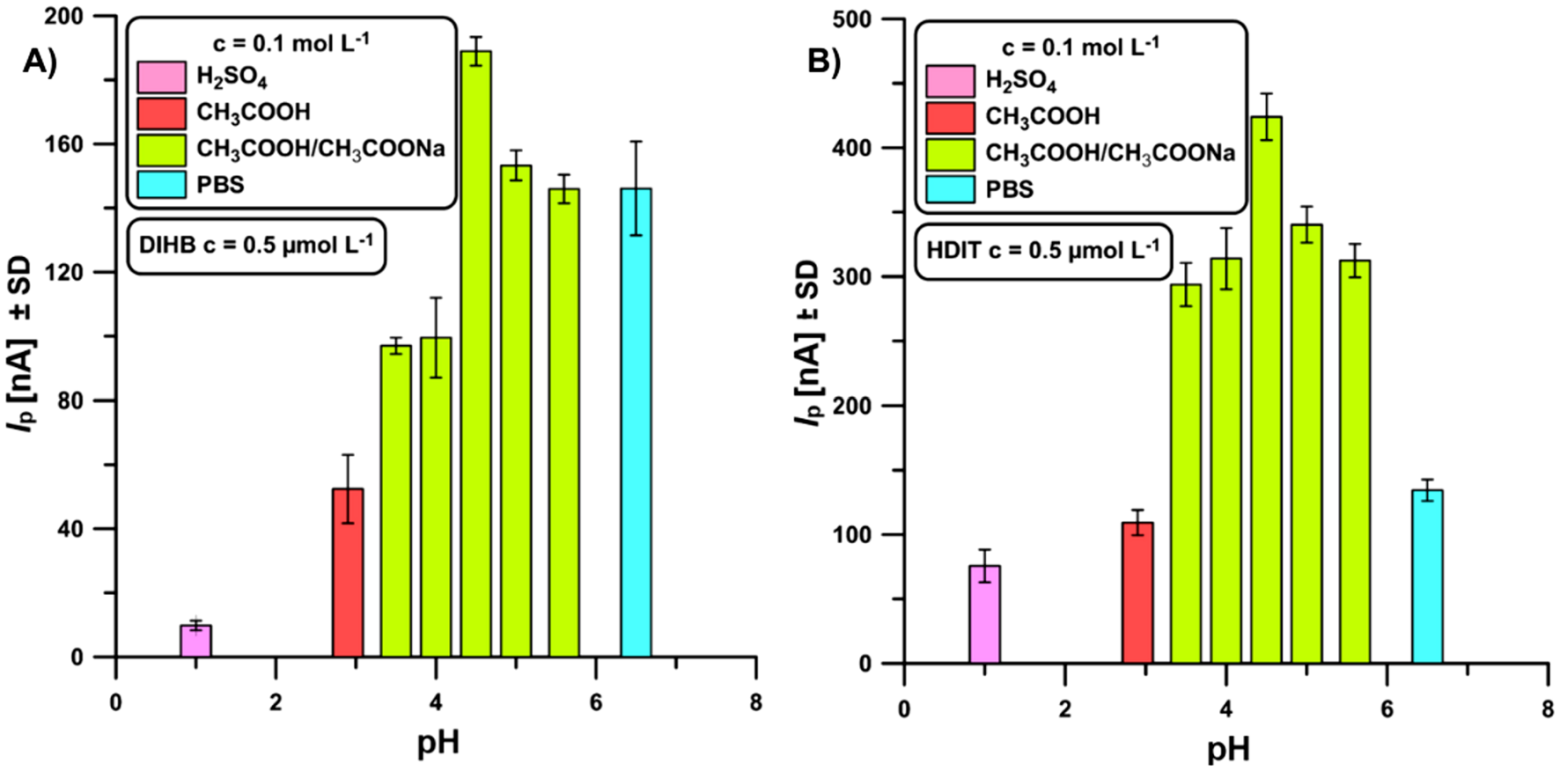
The relationship between the pH of the 0.1 mol L^−1^ supporting electrolyte and the oxidation peak current of 0.5 µmol L^−1^ DIHB (**A**) and HDIT (**B**). The DPV parameters: *ν* of 125 mV s^−1^, Δ*E*_A_ of 100 mV, *t*_m_ of 40 ms. The average *I*_p_ values are given with standard deviation (SD, n = 3).

In the subsequent stage of the investigation, the suitability of 0.1 mol L^−1^ acetate buffer solutions at pH of 3.5, 4.0, 4.5, 5.0, 5.6 as the supporting electrolyte was assessed. To achieve it, the possibility of using other solutions, including 0.1 mol L^−1^ H_2_SO_4_, CH_3_COOH and PBS buffer at pH of 6.5 was investigated. The highest signals of 0.5 µmol L^−1^ DIHB and HDIT were obtained for 0.1 mol L^−1^ acetate buffer solutions at pH of 4.5 (Fig. 5). Moreover, the impact of electrolyte concentration on the 0.2 µmol L^−1^ DIHB and HDIT peaks was scrutinised. The peak current intensity of both tested analytes increased with increasing concentration up to 0.125 mol L^−1^. Then the signals dropped. The increase is related to the improvement of the electrical conductivity of the supporting electrolyte. At concentrations higher than 0.125 mol L^−1^, subsequent processes may occur that contribute to a reduction in the efficiency of analytes determination^38^.

**Figure 6:**
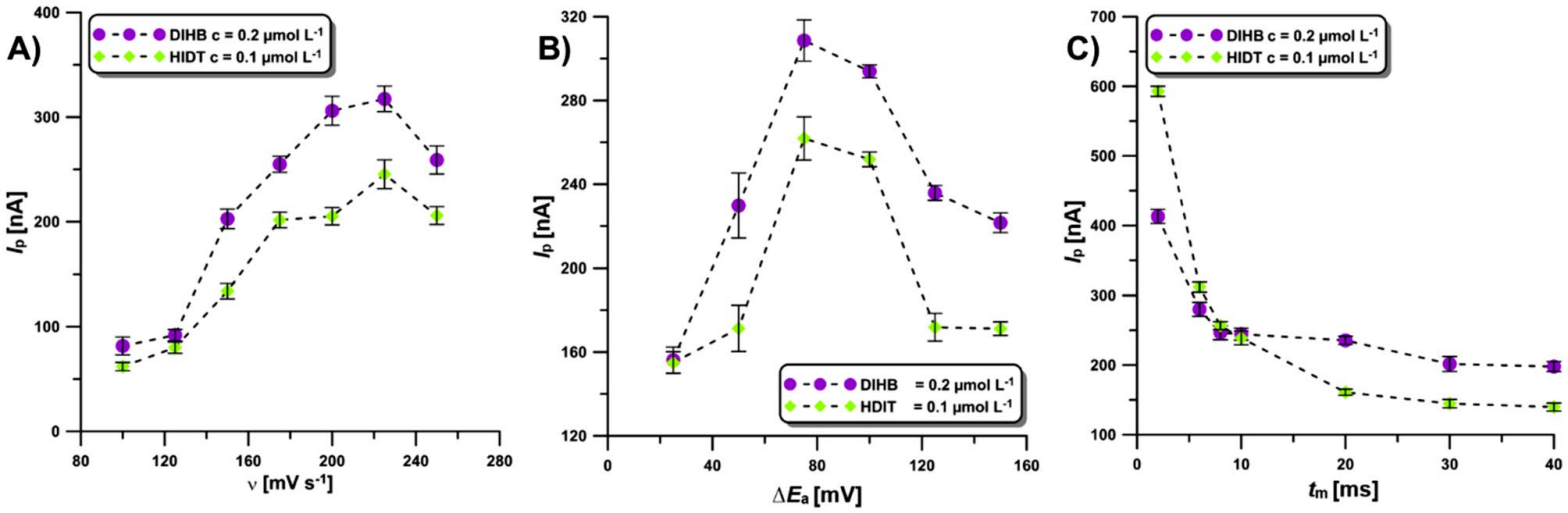
The influence of *ν* (**A**), Δ*E*_A_ (**B**) and *t*_m_ (**C**) on the 0.2 µmol L^−1^ DIHB and 0.1 µmol L^−1^ HDIT *I*_p_ intensity. The average *I*_p_ values are given with standard deviation (SD, n = 3).

In the next stage of the research, the following parameters of the DPV technique were optimised: the scan rate (*υ*), the amplitude (Δ*E*_A_) and the modulation time (*t*_m_). With the increase of *ν*, the *I*_p_ of 0.2 µmol L^−1^ DIHB and 0.1 µmol L^−1^ HDIT increased, reaching the highest intensity at the scan rate of 225 mV s^−1^. Further increasing *ν* resulted in a lower analytical signal (Fig. 6A). Next, the effect of Δ*E*_A_ on the 0.2 µmol L^−1^ DIHB and 0.1 µmol L^−1^ HDIT *I*_p_ intensity was evaluated (Fig. 6B). The *I*_p_ for both analytes reached a maximum value for Δ*E*_A_ of 75 mV. The impact of *t*_m_ was evaluated in the range of 2–40 ms. The maximum DIHB and HDIT *I*_p_ intensity was recorded at 2 ms (Fig. 6C).

### Voltammetric analysis of DIHB and HDIT—calibration graphs, repeatability and reproducibility

**Figure 7:**
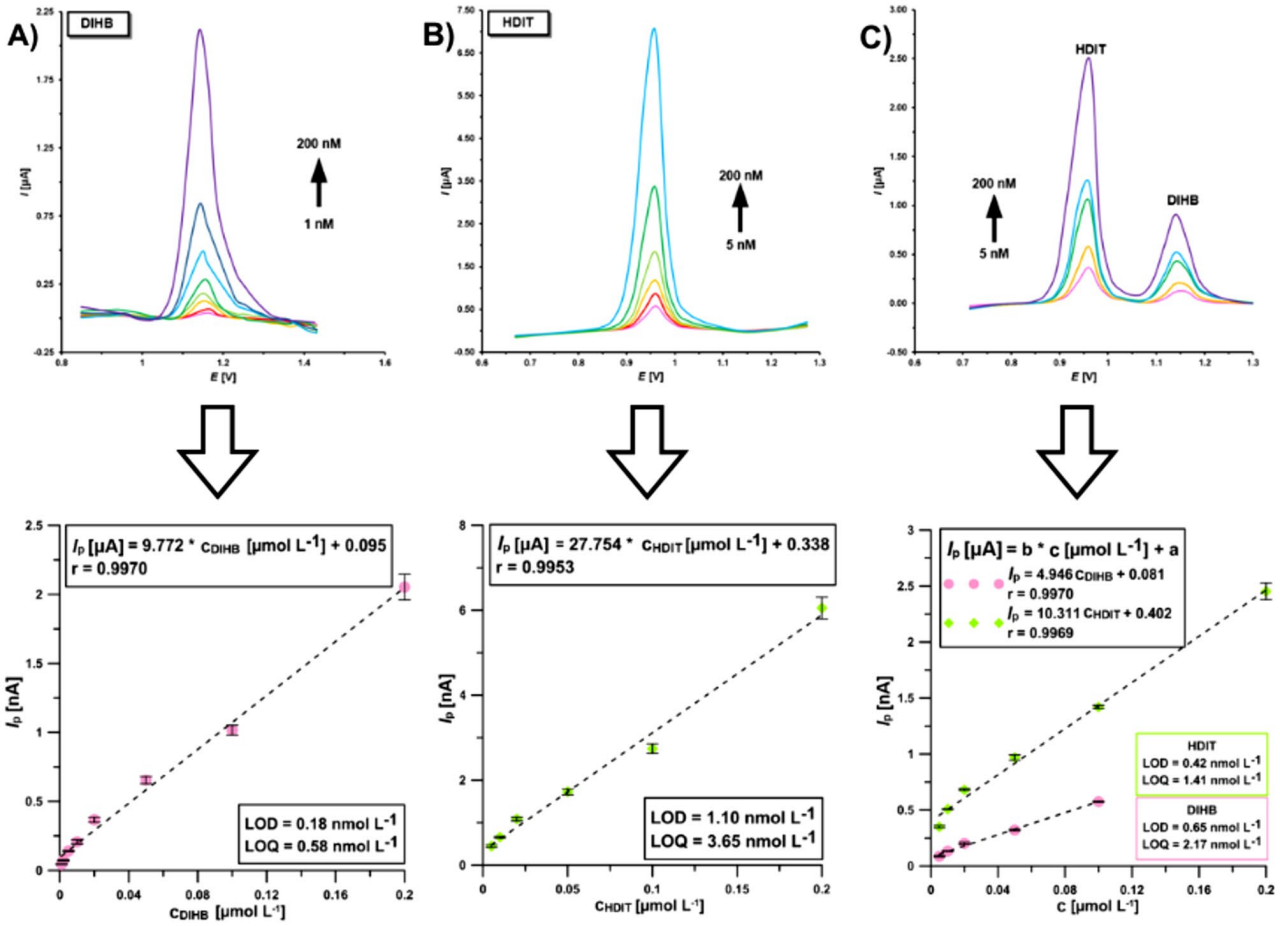
The DPV curves and calibration graphs at the GCE in the presence of increasing concentrations of (**A**) DIHB (1–200 nmol L^−1^), (**B**) HDIT (5–200 nmol L^−1^), (**C**) DIHB (5–100 nmol L^−1^) and HDIT (5–200 nmol L^−1^) in 0.125 mol L^−1^ acetate buffer solution at pH of 4.5. The DPV parameters: *ν* of 225 mV s^−1^, Δ*E*_A_ of 75 mV, *t*_m_ of 2 ms, and the solution mixing time (without applying the potential) of 60 s.

With the DPV parameters optimised for DIHB and HDIT, the calibration curves were constructed in the 0.125 mol L^−1^ acetate buffer solution at pH of 4.5. Figure 7A and B show the DPV curves obtained after individual determination of DIHB and HDIT at the GCE. The linear relationships between the anodic peak current, *I*_p_, and DIHB or HDIT bulk concentrations, were found in the range from 0.001 to 0.2 µmol L^−1^ for DIHB and from 0.005 to 0.2 µmol L^−1^ for HDIT, with an analytical sensitivity of 9.77 and 27.8 µA µmol L^−1^, respectively. The limits of detection (LOD) and quantification (LOQ) determined during individual analysis of DIHB or HDIT were 0.18 and 1.10, 0.58 and 3.65 nmol L^−1^, respectively (calculated as 3 × SD of intercept (n = 3) divided by slope of calibration plot, LOQ was calculated as 3.3(3) × LOD)^39,40^. On the other hand, the linear relationships between *I*_p_ and concentrations of DIHB or HDIT during their simultaneous determination were found in the range from 0.005 to 0.1 µmol L^−1^ for DIHB and from 0.005 to 0.2 µmol L^−1^ for HDIT (Fig. 7C). The LODs and LOQs were 0.65 and 2.17 nmol L^−1^ for DIHB, 0.42 and 1.41 nmol L^−1^ for HDIT, respectively.

Repeatability of the DIHB or HDIT signals was assessed by measuring the peak currents for each point on the calibration curves at the same sensor (i.e., GCE), resulting in the calculated percentage variation coefficient (%RSD) of 5.1% for DIHB and 5.5% for HDIT. The results confirmed that the passivation of the electrode with the HDIT oxidation product does not influence the quantitative analysis of the tested anticancer drug candidates. The reproducibility was tested by measuring 10 times the 0.1 µmol L^−1^ DIHB and HDIT peak currents on three different GCEs. A percentage variation coefficient (%RSD) of 7% for DIHB and 8.1% for HDIT was obtained.

A greenness assessment such as the AGREE metric was calculated. The assessment criteria are taken from the 12 principles of green analytical chemistry (SIGNIFICANCE) and are transformed into a unified 0–1 scale^41^. The final score of 0.8 confirmed the environmental friendliness of the proposed voltammetric procedure.

### Voltammetric analysis of DIHB and HDIT—selectivity and application

The selectivity of the proposed DPV procedure with GCE was examined before analysing the real samples. The possible interferents were selected considering the composition of urine samples (the spiked urine reference material analysis without any prior treatment). Different ions (Fe^3+^, Mg^2+^, Ca^2+^, Cl^−^, PO_4_^3−^, SO_4_^2−^) and organic compounds (urea, creatinine, uric acid, dopamine, adenine) were added to the 0.2 µmol L^−1^ DIHB and HDIT in 0.125 mol L^−1^ acetate buffer solution at pH of 4.5, and the DPV measurements were performed. There was no significant change in the peak currents of DIHB and HDIT (below 10%) in the presence of a 30-fold excess of the tested interferents to the concentrations of analytes. Thus, the results suggested that the proposed DPV procedure with GCE can accurately determine DIHB and HDIT in real urine samples.

To show the analytical utility of the DPV procedure using the GCE as a sensor, the analysis of urine reference material spiked with known concentrations of DIHB and HDIT, without any prior treatment, was performed. The results (Table 1) revealed an average recovery of 97.5%, demonstrating the satisfactory degree of accuracy of the developed analytical procedure.

**Table 1:** The results of simultaneous DIHB and HDIT determination in the urine reference material.

DIHB concentration [µmol L^−1^] ± SD (n = 3)			
Added	Found DPV	Coefficient of variation^a^ [%]	Recovery^b^ [%]
2.0	0.0197 ± 0.00077	3.9	98.5
4.0	0.0391 ± 0.0012	3.1	97.7

HDIT concentration [µmol L^−1^] ± SD (n = 3)			
Added	Found DPV	Coefficient of variation^a^ [%]	Recovery^b^ [%]
2.0	0.0185 ± 0.00066	3.6	92.3
4.0	0.0406 ± 0.00077	1.8	101.6

^a^Coefficient of variation [%] = (SD × 100)/Found DPV.

^b^Recovery [%] = (Found DPV × 100)/Added.

## Methods

### Analytes and reagents

For current electrochemical research needs, two electroactive molecules (i.e., dimethyl 2-[2-(1-phenyl-4,5-dihydro-1*H*-imidazol-2-yl)hydrazinylidene]butanedioate (DIHB) and 8-(3-chlorophenyl)-2,6,7,8-tetrahydroimidazo[2,1-*c*][1,2,4]triazine-3,4-dione (HDIT) were chosen. DIHB and HDIT have been synthesised according to the procedures reported previously^1,3,4^. The structural determination of these analytes had previously been achieved from their experimental spectroscopic and X-ray diffraction data^1,3–6^. The 1 mmol L^−1^ stock solutions of DIHB and HDIT were prepared daily in *N,N*-dimethylformamide (Sigma-Aldrich, Saint Louis, MO, USA). Working solutions of DIHB and HDIT were prepared daily by dilution of stock solutions in ultra-purified water (> 18 MΩ cm, Milli-Q system, Millipore, UK). Merck reagents (Darmstadt, Germany) were used for preparation of acetate buffer solutions at pH of 3.5, 4.0, 4.5, 5.0, 5.6, H_2_SO_4_, CH_3_COOH and PBS (phosphate buffered saline) at pH of 6.5 as well as standard solutions of Fe^3+^, Mg^2+^, Ca^2+^, Cl^−^, PO_4_^3−^, SO_4_^2−^, urea, creatinine, uric acid, dopamine, and adenine.

The urine reference material (ERM-BB386, bovine urine, European Reference Materials, Geel, Belgium) was spiked with known concentrations of DIHB and HDIT and 100 µL of the sample was added to an electrochemical cell, and next the DPV determinations were performed.

### Apparatus and experimental measurements

The measurements were carried out with a µAutolab potentiostat (Eco Chemie, Netherlands) and run with an electrochemical analysis by GPES 4.9 software. A three-electrode glass cell (10 mL volume) was used during all the electrochemical measurements. The working electrode was a GCE (Mineral, Warsaw, Poland, 1 mm diameter). The GCE was polished with alumina slurries of 0.3 µm during 1 min each, and sonicated in a water bath during 1 min. A platinum wire and an Ag/AgCl, 3 mol L^−1^ KCl were used as counter and reference electrodes, respectively.

In the CV measurements, the *ν* was varied from 0.025 to 1.0 V s^−1^. The DPV measurements of DIHB and HDIT (under the optimised conditions) were performed in the 0.125 mol L^−1^ acetate buffer solution at pH of 4.5. The remaining optimum parameters are as follows: *ν* of 225 mV s^−1^, Δ*E*_A_ of 75 mV, *t*_m_ of 2 ms, the solution mixing time (without applying the potential) before each measurement of 60 s.

## Conclusions

DIHB and HDIT have fully established structures, well-documented pharmacological profiles and favourable thermal and electrochemical properties. Thus, the first analytical procedures allowing their individual as well as simultaneous determination have been developed and presented in this paper. These do not require an expensive measuring equipment and environmentally unfriendly solvents and reagents, which can significantly reduce the generation of waste. The differential pulse voltammetry with an unmodified glassy carbon electrode—as a reusable and environmentally friendly sensor—was utilised for the simple, sensitive and selective individual and simultaneous determination of our possible anticancer drugs. The developed procedure under the optimised conditions demonstrated the broad linear ranges (1–200 nmol L^−1^—DIHB and 5–200 nmol L^−1^—HDIT), and the low detection limits (0.18 nmol L^−1^ for DIHB and 1.1 nmol L^−1^ for HDIT). Additionally, it was successfully applied to the determination of DIHB and HDIT in the spiked urine reference material without any prior treatment. The satisfactory results of recovery studies confirmed the accuracy of the elaborated procedure. Because of the electrochemical activity, high sensitivity, good selectivity, no need to modify the surface, commercial availability, reusable and environmental friendliness, the GCE can be used for a routine analysis of DIHB and HDIT in real urine samples. The electrochemical behaviours of DIHB and HDIT were demonstrated for the first time.

## Data Availability

All data generated or analysed during this study are included in this published article or are available from the corresponding author on reasonable request.
